# CD137 signaling enhances tight junction resistance in intestinal epithelial cells

**DOI:** 10.14814/phy2.12090

**Published:** 2014-08-05

**Authors:** Veronica Gusti, Kaila M. Bennett, David D. Lo

**Affiliations:** 1 Division of Biomedical Sciences, University of California, Riverside School of Medicine, Riverside, California

**Keywords:** Epithelium, lymphotoxin, mucosal barrier, tight junction

## Abstract

Treatment of Caco‐2‐BBe intestinal epithelial cells (BBe) with TNF‐*α* and lymphotoxin‐*β* (LT‐*β*) receptor agonists induced the expression of the TNF receptor superfamily gene *TNFRSF9/CD137*. In the gut, these cytokines are known to be involved in both inflammatory responses and development of organized lymphoid tissues; thus, it was notable that in *CD137*‐deficient mice Peyer's patch M cells lacked transcytosis function. To examine the direct effect of CD137 expression on epithelial cell function independent of other cytokine effects including CD137L triggering, we stably transfected BBe cells to express CD137. CD137 was found at the cell surface as well as the cytoplasm, and confocal microscopy suggested that aggregates of CD137 at the lateral and basolateral surface may be associated with cytoplasmic actin filament termini. Many of the CD137 clusters were colocalized with extracellular fibronectin providing a possible alternative ligand for CD137. Interestingly, we found that CD137‐expressing cells showed significantly higher transepithelial electrical resistance (TEER) accompanied by an increase in claudin‐4 and decrease in claudin‐3 protein expression. By contrast, transfection with a truncated CD137 lacking the cytoplasmic signaling domain did not affect TEER. Finally, CD137‐deficient mice showed increased intestinal permeability upon dextran sodium sulfate (DSS) treatment as compared to control mice. Our results suggest that cytokine‐induced expression of CD137 may be important in enhancing epithelial barrier function in the presence of intestinal inflammation as well as influencing cytoskeletal organization.

## Abstract (precis)

CD137 expression induced in intestinal epithelial cells by inflammatory cytokines causes an increase in transepithelial electrical resistance, in part, by increasing Claudin 4 expression. This may play a role in helping maintain barrier function during intestinal inflammation.

## Introduction

Organized inflammation involves complex regulation of a family of pro‐inflammatory cytokines that consists of lymphotoxin, TNF and its corresponding receptors; these cytokines are known to play a role in the organogenesis of mucosal lymphoid tissues such as Peyer's patches (PP), nasopharyngeal associated lymphoid tissues (NALT), as well as inducible tissues such as isolated lymphoid follicles (ILF) (Togni et al. 1994; Koni et al. 1997; Fütterer et al. 1998; Kiyono and Fukuyama 2004). These cytokines induce NF‐*κ*B‐mediated stromal cell expression of a series of chemokines including CCL19, CCL21, and CXCL13 which facilitate the accumulation of lymphoid cells (Honda et al. 2001; Nishikawa et al. 2003). Accompanying the formation of the lymphoid follicle, the overlying intestinal epithelial cells also undergo changes due to the microenvironment created by the underlying lymphoid immune cells. Thus, the gene profile of these Peyer's patch follicle‐associated epithelial cells (PPFAE) are distinct from neighboring enterocytes. For example, PPFAE have been found to express CCL20, CCL9 (in mice), CCL23 (in human), and RelB; these signals in return maintain the lymphoid follicle homeostasis (Lo et al. 2004; Terahara et al. 2008; Wang et al. 2009). It is also thought that this cytokine‐rich microenvironment is part of the differentiation program of specialized epithelial cell subsets in the FAE, such as M cells and goblet cells. Hence, aside from the TNF receptor/ligand superfamily involvement in PP organogenesis, M‐cell functional and structural development is further specified with other TNF superfamily receptor or ligands such as RANKL and CD137 (Knoop et al. 2009; Hsieh et al. 2010).

The mucosal epithelial cell barrier function requires maturation of the tight junction, a continuous intercellular contact point near the cell apex responsible for paracellular permeability and barrier function (Hartsock and Nelson 2008; Suzuki 2013). The intestinal epithelial tight junction permeability is dynamically regulated by dietary factors as well as interactions between the luminal microbiota and the host immune system (Capaldo and Nusrat 2009; Ulluwishewa et al. 2011). A breach in tight junction integrity can cause acute and chronic intestinal inflammation which in turn elevates the level of cytokines such as TNF‐*α* and IFN‐*γ* (Braegger et al. 1992; Dionne et al. 1997; Hering et al. 2012). Treatment of TNF‐*α* alone or together with IFN‐*γ* is known to elevate expression of members of the TNF receptor superfamily (TNFRI and TNFRII), and to change the expression level and subcellular localization of tight junction proteins (Ma et al. 2004; Wang et al. 2005, 2006). Variation in paracellular permeability among cell and tissue types also can be dependent on the composition of transmembrane tight junctional proteins such as occludin, claudin family, junctional adhesion molecule (JAM) family, and tricellulin (Hartsock and Nelson 2008; Suzuki 2013).

Like other TNF receptor superfamily members, CD137 (also known as 4‐1BB or Tnsfr9) and its ligand, CD137L (4‐1BBL or Tnfsl9) induce activation of various cellular responses such as proliferation, survival, and apoptosis (Lee and Kwon 2006), but studies on CD137 signaling have mainly focused on its role in immune cells. Yet, in endothelial cells, CD137 expression upon TNF‐*α* induction facilitates monocyte extravasation (Quek et al. 2010), and we recently reported a strong CD137 induction in TNF‐*α* and lymphotoxin‐treated intestinal epithelial cells (Wang et al. 2009; Hsieh et al. 2010). CD137 function and signaling in intestinal epithelial cells has not been extensively explored; this is surprising considering CD137 is known to bind to extracellular matrix components such as laminin, collagen I, and fibronectin (Chalupny et al. 1992; Loo et al. 1997), in addition to its more commonly known ligand CD137L.

Since CD137 is important in M‐cell functional maturation, including basolateral pocket formation (Furuse et al. 1999), we sought more detailed information on any potential function of CD137 in intestinal epithelial cells, including possible increased interaction with cytoskeletal proteins and accompanying functional changes. We found that CD137 expressed in epithelial cells appeared capable of interacting with cytoplasmic actin filaments at the lateral and basolateral side of the polarized cells, and also increased transepithelial electrical resistance associated with tight junction changes. These results suggest that CD137 may have multiple complex functions depending on its cellular context.

## Materials and Methods

### Cell culture

C2BBe1 (BBe, clone of Caco‐2, Caco2‐BBe, ATCC# CRL‐2102) a colon carcinoma cell line was grown for 3 weeks to form a polarized monolayer in Advanced Dulbecco's Modified Eagle's Medium (ADMEM‐Life Technologies, Grand Island, NY) supplemented with 10% fetal bovine serum (Biowest, Kansas City, MO), 10 mmol/L HEPES, and 1X penicillin‐streptomycin‐glutamine (Cellgro Mediatech, Manassas, VA). Media were changed every 3 days.

### Full‐length CD137 plasmid construction (pCAG‐CD137)

The CD137 open reading frame was PCR amplified from total cDNA from 48‐h cytokine‐treated BBe cells, as described in Wang et al. (2009). The forward primer spanned the start codon with flanking 5′ AgeI site and the reverse primer spanned the stop codon with flanking 3′ NotI site. The PCR fragment was digested and ligated into AgeI‐NotI sites in pCAG‐eGFP‐IRES‐Puro replacing the eGFP open reading frame (ORF) (Liew et al. 2007). Ligated DNA plasmid was transformed into *E. coli*. pCAG‐CD137‐IRES‐Puro (pCAG‐CD137) plasmid DNA was screened, sequenced, and compared with the CD137 nucleotide sequence from NCBI (GI:315259099).

### CD137 extracellular domain plasmid construction (pCAG‐CD137xtGFP)

The extracellular domain of CD137 was determined according to annotation in Uniprot (#Q07011). The potential cytoplasmic domain was from amino acid (aa) 214–255. The same forward primer used in pCAG‐CD137 was paired with a new reverse primer with flanking 3′AgeI site (CD137xtR‐AgeI: 5′‐CTT GCT CAC CAT GGT GGC GAC CGG TTT TCT GCC CCG TTT AAC AAC AGA GAA ACG GAG CGT‐3′). The primer pair amplified 1‐218aa out of 255aa. Amplified PCR fragments were digested and ligated into AgeI sites in pCAG‐EGFP‐IRES‐Puro right before the EGFP‐ORF. The ligated DNA plasmid was transformed into *E. coli*. pCAG‐CD137extracellular‐GFP‐IRES‐Puro (pCAG‐ CD137xtGFP) plasmid DNA was screened, sequenced, and compared with the CD137 nucleotide sequence from NCBI (GI:315259099).

### Stable transfectant clone generation

Purified DNA plasmids were transfected into BBe using Amaxa nucleofactor kit T according to the manufacturer's optimized protocol for Caco‐2 cell. Transfected cells were allowed to recover for 2 days without antibiotic selection and 10 *μ*g/mL puromycin was added to the media on day 3. Transfected cells were grown under selection for the next 7 days before being clonally seeded via limit dilution. Clones were selected based on the abundance of CD137 mRNA. CD137ORF‐F and CD137ORF‐R were used as screening primers which amplified a portion of the CD137 extracellular domain.

### Transepithelial electrical resistance (TEER) measurements

Cells, 0.5 × 10^6^, of BBe or its CD137 clones were seeded, grown as a monolayer, and TEER was measured for up to 4 weeks post seeding on 3.0‐*μ*m pore–12‐well transwell–1.12 cm^2^ area polycarbonate membrane transwells (Costar#3402). BBe complete media were added to both the top and bottom of the transwell insert. TEER was measured using EVOMX Epithelial Voltohmmeter (World Precision Instruments, Sarasota, FL). Before TEER measurements were taken, the microelectrodes of the EVOMX Voltohmmeter were washed in 70% ethanol and equilibrated in culture media. Microelectrodes were placed on both sides of the transwell insert. The cell monolayer electrical resistance was determined by subtracting the TEER reading of the transwell with the cell monolayer from the blank transwell without the cell monolayer. Each individual experiment was run in triplicate wells and all measurements were read three times at different microelectrode straddling positions. Experiments were repeated three times for biological replicates.

### Immunohistochemistry and confocal microscopy

Cells, 1.0 × 10^5^, were seeded and grown as a monolayer on 8‐well chambered slides (LAB‐TEK) for 3 weeks. Cells were washed with PBS and fixed with one of the following steps: (1) preextracted with 0.2% TritonX/PBS on ice followed by 1% paraformaldehyde/PBS for 15 min at RT; (2) fixed directly with 1% paraformaldehyde/PBS for 15 min at RT; (3) fixed directly with 100% methanol for 5 min on ice; and (4) fixed directly with 100% Acetone for 5 min on ice. Cells were permeabilized with 0.5% Tween/PBS and blocked in 0.1% Tween/casein/PBS before primary antibody staining. Tissue postfixation was done with 4% paraformaldehyde/PBS and mounted with Prolong Gold antifade reagent with DAPI (Life Technologies). Images were obtained using a spinning disk BD CARVII Confocal Imager (BD Biosystems, San Jose, CA) on a Zeiss Axio Observer inverted microscope (Carl Zeiss Microscopy, Thornwood, NY) at 63X objective lens controlled by MetaMorph imaging software (Molecular Devices, Sunnyvale, CA). Image Z resolution was further optimized with Volocity software (PerkinElmer, Waltham, MA). For non‐Z‐stacked higher resolution images, a Leica SP5 inverted laser confocal micorscope was used. Antibodies used: Anti‐claudin 3(#ab15102, AbCam, Cambridge, MA), anti‐claudin4 (#329400, Life Technologies), Anti‐human CD137 (#552533, BDBioscience), phalloidin‐Alexa 647(A22287, Life Technologies).

### Western blot

Cells, 0.5 × 10^6^, were seeded and grown as mature monolayer on 6‐well cluster plates for 3 weeks. Monolayers were harvested with a cell scraper in 200 *μ*L of RIPA buffer and 1× Halt proteinase inhibitor cocktail (Thermo Fisher Scientific, Waltham, MA). Cells were sonicated with 2‐ to 5‐sec pulses and placed on ice. Approximately 20 μg of samples was ran on 4–12% or 12% Bis‐tris gel (Life Technologies) and transferred onto nitrocellulose membranes for antibodies hybridization. Membranes were scanned using an Odyssey imaging system (LI‐COR) and the detected protein band was quantified using the ImageJ software (National Institutes of Health, Bethesda, MD). The quantified band was statistically analyzed using two‐tailed, unpaired, Student's *t*‐test.

### Real‐time PCR

Cell monolayers were cultured as mature monolayers on six‐well cluster plates for 3 weeks and RNA was extracted using TRIzol reagent (Life Technologies). Three or 5 *μ*g of total RNA in 20‐*μ*L total reactions was reversed transcribed into cDNA using the Superscript III first‐strand synthesis system (Life Technologies). One microliter of the cDNA was used to detect real‐time PCR products using 2× SYBR Green master mix (Bio‐Rad, Hercules, CA) and the Biorad CFX96 qPCR machine (Bio‐Rad). Human GAPDH was used as a reference gene for all qPCRs. The comparative ΔΔCT method was used to determine the relative amount of gene expression between BBe and its derivative clones. The quantified band was statistically analyzed using two‐tailed, unpaired, Student's *t*‐test. Human primers used are as follows: EPCAM‐F 5′‐TCC TGA CTG CGA TGA GAG CG‐3′, EPCAM‐R 5′‐CCT TCT GAA GTG CAG TCC GC‐3′, Villin‐F 5′‐ ‐3′, Villin‐R 5′‐ ‐3′, Cldn 3‐F5′‐ CGA GAG CGT ATG GAG CCG AG‐3′, Cldn3‐R 5′‐ CGT GAT GAT GTT GCT GCC GA‐3′, CD137ORF‐F 5′‐ATG CAG GCA GTG TAA AGG TGT‐3′, CD137ORF‐R 5′‐ CCA CGT CCC TCT CCT TCG TC‐3′. For *GAPDH, CCL20, CD137UTR (Tnfrsf9), GP2* primers sequences were as published in Wang et al. (2009).

### Scanning electron microscopy

Cell monolayers were grown on glass‐bottom Petri dishes for 3 weeks and fixed with 2.5% glutaraldehyde (Ted Pella, Redding, CA) solution for 30 min. Samples were then washed in ddH20 and 4% osmium tetroxide (Ted Pella) was added for 30 min. Samples were gradually dehydrated in 25, 50, 75, 90, and 100% ethanol for 5‐min intervals. Dehydrated samples remained immersed in 100% ethanol until critical point drying was performed using Balzar's Critical Point dryer (CPD) according to the manufacturer's protocol. Dried samples were then mounted onto SEM pin stub mounts (Ted Pella) with carbon‐coated conductive tape and sputter coated with platinum/palladium for 60 sec (Sputter Coater Cressington 108 auto; Cressington Scientific Instruments, Watford, UK). The apical surface of cells was then viewed using XL‐30 FEG Scanning electron microscope at 10 kV.

### DSS‐induced acute colitis in mice and intestinal permeability assay

Eight‐ to twelve‐week‐old CD137^−/−^ on the BALB/c background and wild‐type (WT) BALB/c mice were administered 5% Dextran Sodium Sulfate (DSS, MW 36,000–50,000, MP Biomedicals, Santa Ana, CA) in drinking water ad libitum for 7 days; noncolitis control mice were given tap water. At day 7, mice were fasted and 5% DSS was replaced with tap water. At day 8, intestinal epithelial cell integrity was assessed: mice were gavaged with 60 mg/100 g of 30 mg/mL fluorescein isothiocyanate (FITC)‐dextran (4 kDa, Sigma‐Aldrich, St. Louis, MO) in 1XPBS. Blood was collected retro‐orbitally 4 h after gavage, and plasma was collected by centrifugation at 8000 g for 10 min at 4°C. Plasma was diluted with equal amount of 1XPBS. 100 *μ*L of diluted plasma was added in duplicate to a clear 96‐well plate (Costar‐ELISA plate, Corning, Tewksbury, MA). Fluorescence was detected at excitation wavelength 360 with emission wavelength 485 nm on Molecular Devices SpectraMax M2e plate reader. The concentration of fluorescein was determined using serially diluted samples of the tracer in 1XPBS as a standard. Fluorescence images of mice given FITC‐dextran were acquired using an iBox Explorer imaging microscope (UVP). All mouse studies were done according to institutional IACUC protocol approval and NIH guidelines.

## Results

### Overexpression of CD137 did not affect FAE and M‐cell‐related gene expression and microvilli formation

**Figure 1.**
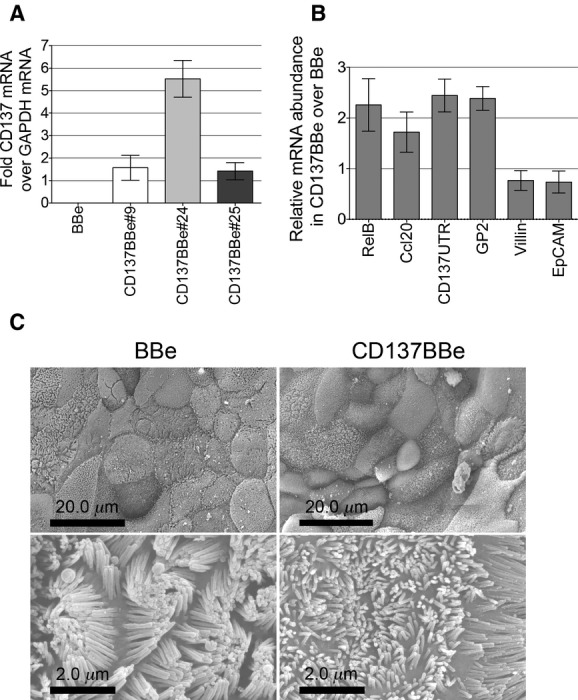
Expression of transfected CD137 did not induce M cell and follicle‐associated epithelium gene expression or affect microvilli formation. (A–B): Real‐time PCR results for CD137 (A) and other epithelial genes (B) of mature monolayers are shown as the average fold induction from three independent experiments (mean ± SEM). (C) Scanning electron micrograph of the apical microvilli of matured BBe and CD137BBe monolayers.

Cytokines have been shown to induce changes in multiple cellular processes including cell proliferation, apoptosis, metabolism, and the secondary signaling that can indirectly affect cellular function (Lee and Kwon 2006; Capaldo and Nusrat 2009; Rock et al. 2011; Osborn and Olefsky 2012). To examine the direct effect of *CD137* expression on epithelial cell function separately from other cytokine effects (including CD137L triggering), we stably transfected Caco‐2BBe (BBe) cells to express CD137. To minimize cellular heterogeneity among the transfected cells studied, we used limit dilution to isolate clones for further study. This would help insure that measurements of epithelial functions such as barrier function, signaling, and transepithelial electrical resistance would not be undermined by variant cells that have lost epithelial functions. We first examined the level of *CD137* mRNA expression by quantitative PCR and the formation of the microvilli of the stable CD137Caco‐2BBe (CD137BBe) clones after 3 weeks of culture. Since *CD137* is not actively expressed in nontransfected BBe, we measured the relative abundance of the *CD137* mRNA against *GAPDH* mRNA by amplifying the a portion of the extracellular domain of the *CD137* coding sequence. We found that the expression of *CD137* in the three clones was at least comparable with the *GAPDH* mRNA abundance, suggesting a high expression level compared to the nontransfected BBe (Fig. 1A). Since our results proved to be consistent among the differen clones, for most of the remaining studies we used the CD137BBe#24 clone to represent *CD137* overexpression in BBe.

Next, we examined the effect of CD137 overexpression on FAE and M‐cell‐related gene expression since these genes were upregulated together with endogenous *CD137* in BBe in the presence of cytokine treatment as shown in Wang et al. (2009); endogenous *CD137* transcripts were measured by amplifying the *CD137* untranslated region (CD137UTR). Using the primer sequences described in Wang et al. (2009), we found that the stable overexpression of CD137 did not significantly change the mRNA expression level of FAE and M‐cell‐related genes such as *RELB*,* CCL20*, endogenous *CD137*, and *GP2* compared to the nontransfected BBe (Fig. 1B). To ensure that there is no autosignaling through expression of both CD137 and CD137L, we verified that the *CD137L* transcript was undetectable by real‐time PCR. We also checked the mRNA level of villin and *EPCAM* to examine the potential role of CD137 in microvilli formation or epithelial adhesion and we found no difference in transcription level between the CD137‐transfected cells and controls (Fig. 1B). To examine the role of CD137 on the polarization of mature BBe monolayers, we started by looking at microvilli formation; moreover, one hallmark of M‐cell development is the absence of apical microvilli. Using scanning electron microscopy (SEM), we found that the overexpression of CD137 in BBe did not inhibit microvilli formation (Fig. 1C). In addition, immunostaining for ZO‐1, Occludin, E‐Cadherin, and JAM‐A showed no effect on the distribution or expression of these tight junction proteins (not shown). These results confirm that expression of CD137 in epithelial cells alone did not alter cellular gene expression or morphology, so it is not by itself likely to be responsible for the M‐cell developmental program, at least without triggering by a known ligand or other influence.

### Increased TEER on CD137BBe stable clones

**Figure 2.**
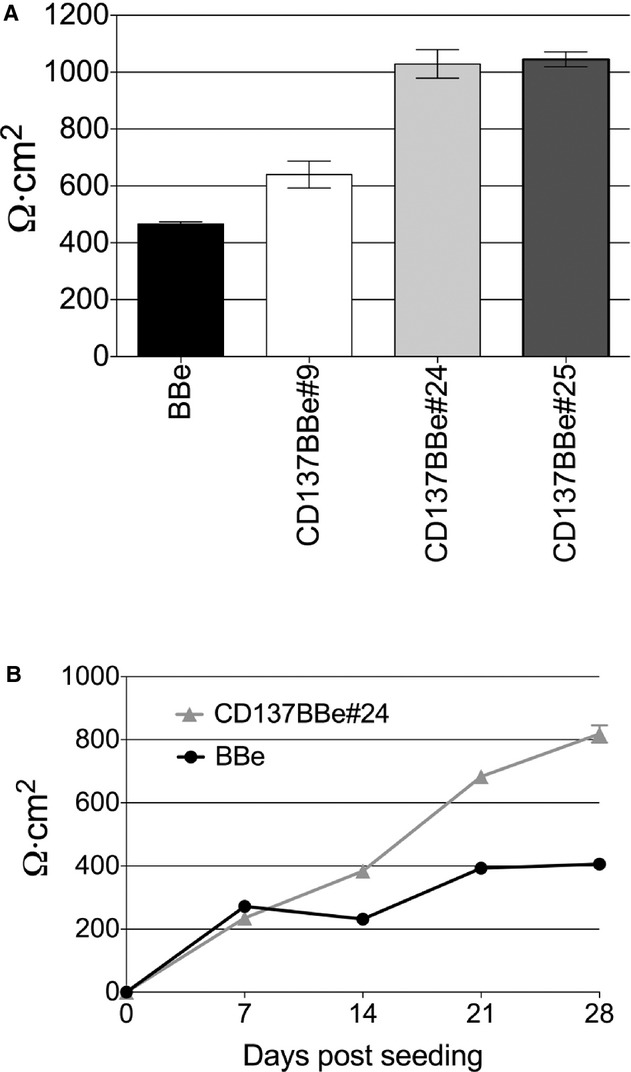
CD137‐expressing cell monolayers show increased TEER. (A) TEER reading of three CD137BBe clones at 3 weeks compared to control BBe. (B) Progression of TEER reading on CD137BBe and BBe up to 4 weeks post seeding. The TEER reading shown is representative of three independent experiments. Data are plotted as the average resistance of three independent transwells in a single experiment (mean ± SEM).

To examine tight junction maturity, we tested the electrical resistance of the cell monolayer on the third week post seeding. We compared three of the CD137BBe clones with nontranfected BBe and found that all of the CD137BBe stably transfected clones had a significantly higher tight junction resistance reading compared to the control (Fig. 2A). However, when compared to the abundance of *CD137* mRNA transcripts (Fig. 1A), the TEER reading did not proportionally correspond to CD137 transcript levels, suggesting an indirect interaction between factors affecting the tight junction protein composition and CD137.

To examine the progression of the tight junction electrical resistance, we followed the TEER reading of the cell monolayer for up to 4 weeks post seeding. The BBe and CD137BBe monolayer had comparable tight junction electrical resistance maturity rates during the first week of growth (Fig. 1B). However, on the second week, the BBe monolayer tight junction electrical resistance maturity rate was lower compared to CD137BBe (Fig. 1B). Hence, it resulted in a significant difference between the electrical resistance readings by the third week of growth post seeding. Both cell monolayers have a lower rate of TEER increase after the third week post seeding, suggesting that tight junctions in both cell monolayers have matured. By the end of 4 weeks of culture, we found that most cultures have reached their maximal TEER, with the CD137BBe at significantly higher TEER than controls (see also below). This result implies that CD137BBe and BBe have a similar rate of tight junction maturation; however, the higher TEER in the 137BBe cell lines suggest differences in the final composition of the tight junction.

### Claudin‐3 were decreased and claudin‐4 were increased in the presence of CD137

**Figure 3.**
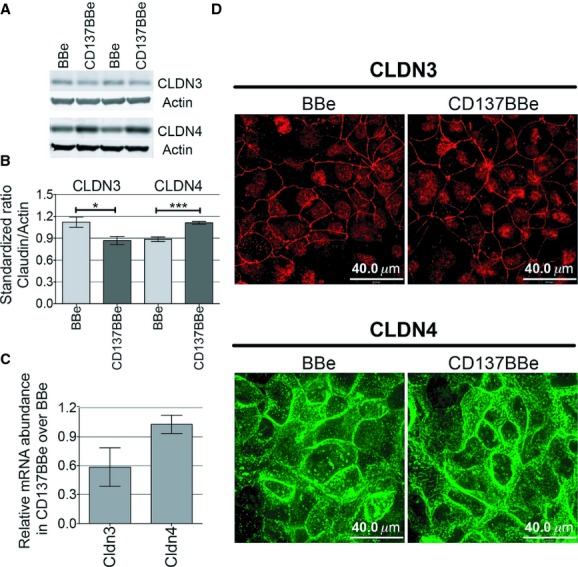
CD137BBe show increased claudin‐4 and decreased claudin‐3 protein expression compared to BBe. (A) Immunoblot of total CLDN3 and CLDN4 expression in CD137BBe compared to control BBe. The blot shown includes two of five biological replicate experiments. (B) Quantification of the claudin immunoblot from five independent experiments shown as the average of relative abundance against actin (mean ± SEM) (Mann–Whitney test results: *P < 0.05; ***P < 0.0005). (C) Real‐time PCR results on matured monolayers are shown as the average of relative abundance against GAPDH from three independent experiments (mean ± SEM). (D) Fluorescence confocal images of CD137BBe and BBe stained with CLDN3 (acetone fixed) and CLDN4 (methanol fixed).

Modulation of claudin expression has been known to affect tight junction integrity, measured as differences in TEER readings (Van Itallie et al. 2001; Colegio et al. 2003; Takehara et al. 2009; Suzuki 2013). To examine claudin expression in CD137BBe, total cell lysates of the mature monolayer were assayed for total claudin protein and mRNA levels. We found that CLDN3 protein expression in CD137BBe was lower, while CLDN4 protein expression was higher compared to the nontransfected BBe via western blot (Fig. 3A and B). We also checked for claudin family mRNA abundance and found that there was no statistically significant change in *CLDN4* mRNA transcript levels, although there were lower *CLDN3* mRNA transcript levels up to 3‐fold (Fig. 3C). There were no significant changes in the distribution of CLDN3 and CLDN4 cellular expression by immunofluorescence staining (Fig. 3D). Not all claudins are expressed in the intestine (Fujita et al. 2006), so we tested the abundance of claudin‐1, 2, 5, 7, 8, 12, 13, 15 mRNA and found that there was less than a twofold change in these genes compared to BBe (data not shown). We used western blot and immunohistochemistry to examine CLDN1, 5, and 7 protein levels as well as their cellular distribution in CD137BBe compared with BBe, and did not detect any significant differences (data not shown). Other tight junction proteins such as JAM‐A, ZO‐1, and occludin also did not show any transcriptional differences (less than twofold difference; data not shown).

### CD137‐mediated increase in TEER is not dependent on NF‐kB and ERK1/2 signaling

**Figure 4.**
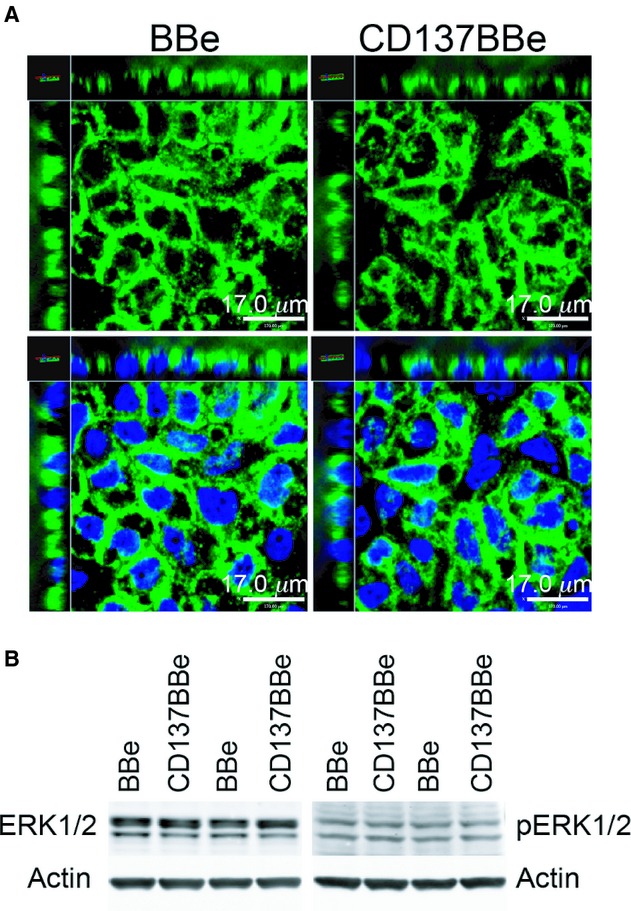
CD137‐mediated TEER increase in CD137BBe is not associated with persistent NF‐*κ*B and ERK1/2 signaling. (A) XYZ fluorescence confocal images of CD137BBe and BBe fixed with methanol and stained with anti‐NF‐*κ*B‐p65 (green) and nuclei (DAPI‐blue). (B) Immunoblot of CD137BBe and BBe total cell lysate for ERK and phospho‐ERK. The blots show two of five independent experiments.

Previous studies of CD137 in T cells have shown that triggering of CD137 activates NF‐kB and ERK that accounts for T‐cell survival and clonal expansion (Arch and Thompson 1998; Jang et al. 1998; Saoulli et al. 1998; Lee et al. 2002; Sabbagh et al. 2008, 2008). In addition, ERK1/2 MAPK downstream signaling has been suggested to reduced claudin‐4 protein expression in porcine intestinal barrier (Pinton et al. 2010). In view of the differences between T cells and epithelium, we examined whether activation of NF‐kB and ERK signaling of CD137 in intestinal epithelial cells in the absence of CD137L‐mediated triggering accounts for the increase of TEER reading in CD137BBe. We looked for NF‐kB p65 fluorescence staining colocalization in the CD137BBe cell nuclei and found that p65 was sequestered in the cytoplasm of the CD137BBe, not in the nuclei (Fig. 4A). Moreover, we found that there was no significant difference in the expression of both phospo‐ERK1/2 and total ERK1/2 in the CD137BBe total cell lysate compared to BBe (Fig. 4B). CD137 is also known to transduce signals through other mitogen‐activated protein kinase pathway, JNK and p38 protein kinases (Saoulli et al. 1998; Cannons et al. 1999, 2000; Watts 2005). However, as with most signaling studies, CD137 signaling activation analysis was done upon induction by acute antibody agonist or CD137L, which result in downstream phosphorylation changes in <1 h (Saoulli et al. 1998; Cannons et al. 1999, 2000; Sabbagh et al. 2008). Since our overexpression model did not rely on acute induction by agonists, changes in MAPK pathway phosporylation as well as NF‐kB nuclear translocation might be unable to represent activation; indeed, while evidence for signaling is easier in the acute setting (e.g., a few hours), signals such as nuclear translocation of NF‐kB are known to recover within several hours or days, so long‐term chronic stimulation may be difficult to demonstrate under these conditions. Thus, considering the persistent localization of CD137 with ECM components, two possibilities explain the absence of evidence for acute signaling: the capture of cell surface CD137 by ECM binding may act to prevent signaling by CD137, or alternatively, ECM binding induces a chronic signaling state with far more subtle indicators of downstream effects.

### The signaling domain of CD137 is required for increased tight junction electrical resistance

**Figure 5.**
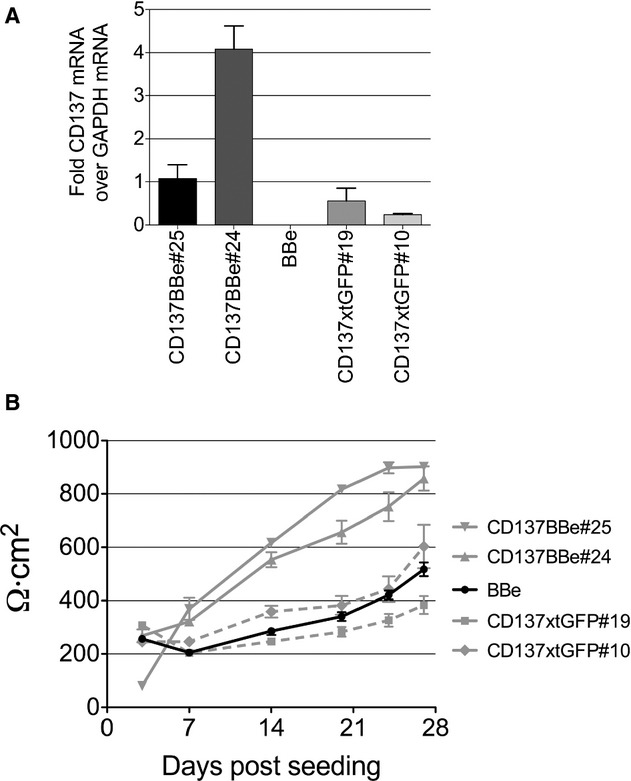
The CD137 signaling domain is required for the TEER increase in 137BBe cells. (A) Real‐time PCR results are shown as the average of relative abundance of truncated CD137xtGFP and full‐length CD137BBe transcripts against GAPDH from three independent experiments (mean ± SEM). (B) TEER reading of CD137xtGFPBBe clones through 4 weeks post seeding compared to CD137BBe and control BBe. The TEER reading shown is representative of three independent experiments. Data are plotted as the average resistance of three independent transwells in a single experiment (mean ± SEM).

To test whether the signaling domain of CD137 is important for the increase in tight junction resistance, we established stable clones overexpressing CD137, in which its signaling domain (214–255aa) had been replaced with GFP (CD137xtGFP); the clone expresses 1‐218aa instead of the full‐length 255aa. We selected the clones by quantifying the extracellular *CD137* mRNA level against the abundance of GAPDH mRNA (Fig. 5A). We found that without the signaling domain, the TEER reading of the three CD137xtGFP monolayer clones had similar electrical resistance compared to the nontransfected BBe (Fig. 5B). This suggests that the signaling domain is necessary for the increase of the TEER observed in the overexpression clones.

### CD137 is associated with fibronectin and actin filament termini

Two previous studies suggested that CD137 interacts with extracellular matrix (ECM) components such as laminin, fibronectin, and collagen I (Chalupny et al. 1992; Loo et al. 1997). Our immunofluorescence staining shows that CD137 aggregates at the basolateral side of the CD137BBe monolayer and colocalizes with fibronectin. Our data not only confirmed the earlier observations but it also suggests that interaction between CD137 and ECM, independent of CD137L, may be related to regulation of tight junction resistance.

**Figure 6.**
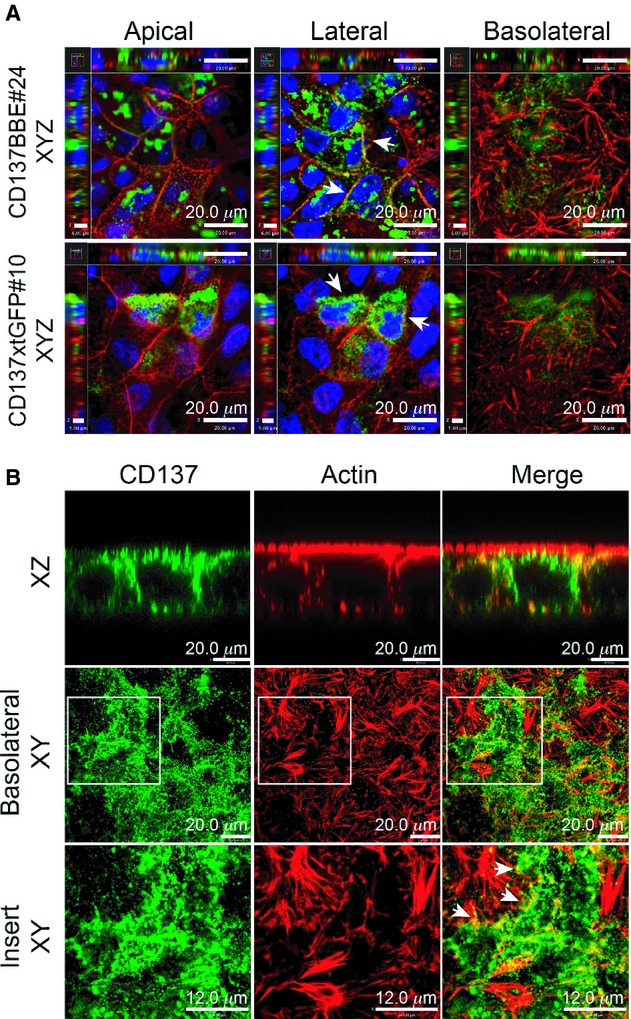
CD137 aggregates at the plasma membrane are also associated with actin filaments. (A) XYZ fluorescence confocal images of monolayers of full‐length CD137BBe#24 and truncated CD137xtGFP#10 clones, preextracted with 0.2% Triton‐X/PBS, fixed with paraformaldehyde, and stained anti‐CD137 (green), phalloidin‐actin (red), and DAPI‐nuclei (blue). The arrows show CD137 association with cortical actin and plasma membrane with the full‐length clone while the truncated CD137 protein was found mainly within the cytoplasm. (B) confocal images of the CD137BBe#24 clone, showing basolateral clusters of CD137 (green) associated with actin filament (red) termini (inset – arrows).

Since it was striking in CD137KO mice that M cells lacked basolateral pockets occupied by B lymphocytes (Hsieh et al. 2010), we noted that there may be a role for CD137 in cytoskeletal reorganization. In the transfected BBe cells, we found that the CD137 was expressed both in the cytoplasm as well as on the cell surface, where it was distributed along the cell membrane. In addition, previous studies suggested that TNFRSF/TNFLSF gene family members, including CD137/CD137L, might be activated by trimerization (Wyzgol et al. 2009; Won et al. 2010); based on our fluorescence staining we noted CD137 clusters on the lateral and basolateral side associating with actin filaments (Fig. 6). By comparison, the truncated CD137 did not localize at the cell membranes as consistently, nor were there clear associations with the ends of actin filaments (Fig. 6).

**Figure 7.**
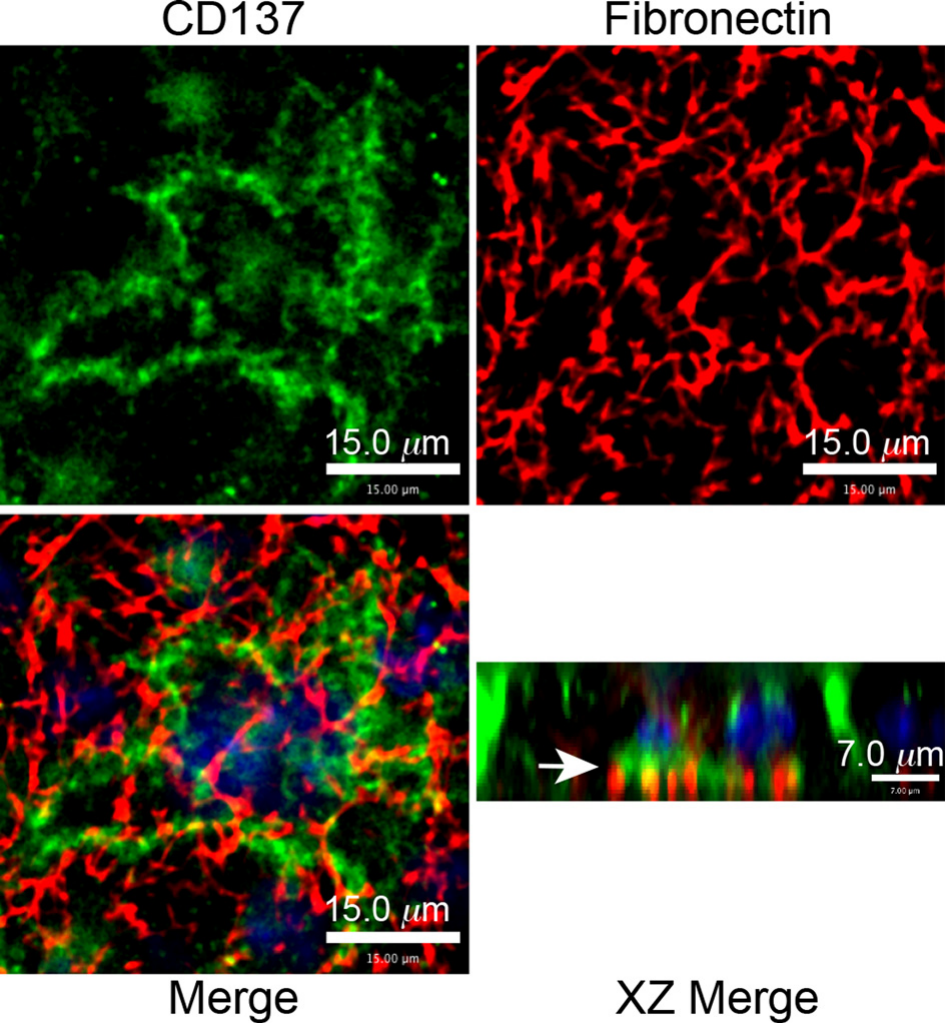
CD137 at basolateral side is associated with extracellular fibronectin. Fluorescence confocal images of CD137BBe at the basolateral side fixed with paraformaldehyde and stained with anti‐fibronectin (red), anti‐CD137 (green), and nuclei (DAPI‐blue). The arrow shows the association of CD137 with fibronectin at the basolateral side.

The clustering of CD137 at the cell membrane suggests that CD137 signaling might be activated by ligands at the lateral and basolateral surface of the epithelial cells, likely to be extracellular matrix components. One candidate for this extracellular component is fibronectin, as there some apparent colocalization of basolateral CD137 with fibronectin especially at cell junctions (Fig. 7).

### CD137‐deficient mice and mucosal barrier function

The in vitro effect of CD137 expression on epithelial tight junctions suggested that the induction of CD137 in vivo might be important in maintaining mucosal barrier function, especially in the face of intestinal inflammation. The hypothesis is that inflammatory cytokines would induce CD137 which in turn could boost tight junction barrier function, promoting barrier integrity during epithelial healing. To test this, we gave a series of control and CD137‐deficient mice a course of DSS in water for 7 days to induce intestinal inflammation. On day 8, dextran‐FITC was given orally to assess intestinal permeability, and the levels of fluorescent signal in the blood were determined.

**Figure 8.**
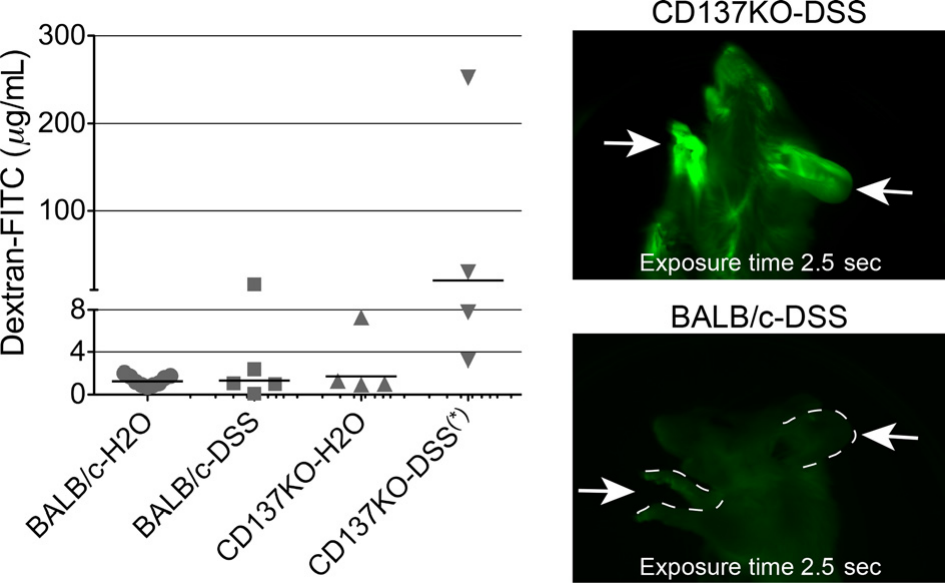
CD137 deficiency results in an impaired mucosal barrier in the face of DSS‐induced inflammation. Wild‐type BALB/c and BALB/c‐backcrossed CD137 knockout mice were given a 7‐day course of DSS, then tested for intestinal permeability to orally administered FITC‐dextran. The figure shows serum FITC levels in mice; images on the right show fluorescence whole body imaging, with strong green fluorescence evident in exposed skin of the paws and ears of the CD137 knockout mouse but not the control (arrows).

As shown in Fig. 8, mice deficient in CD137 was found to have, on average, a higher level of dextran‐FITC in the blood, and this was evident even in fluorescence imaging of the whole animal. There was a large range in fluorescence values in the CD137‐deficient group, but the difference from controls was significant (Mann–Whitney one‐tailed *P* value was 0.03), and the mice appeared more severely affected; one CD137‐deficient mouse could not be tested due to premature death.

## Discussion

Several studies suggested that TNF‐*α* causes a TEER decrease and paracellular permeability increase in mucosal epithelium, although these studies were mainly performed in vitro by treating intestinal epithelial cell monolayers with TNF‐*α* with and without IFN‐*γ* (Marano et al. 1998; Fish et al. 1999; Schmitz et al. 1999; Zolotarevsky et al. 2002; Ma et al. 2004; Prasad et al. 2005; Wang et al. 2005, 2006; Li et al. 2008). Moreover, these studies usually assayed the monolayer within 24–72 h and rarely extended beyond 7 days post treatment (Marano et al. 1998). Since TNF‐*α* is associated with chronic inflammatory bowel disease (Braegger et al. 1992; Dionne et al. 1997), a short period of TNF‐*α* treatment might misrepresent chronic intestinal inflammation. In addition, a study in endothelial cells using pharmacological inhibitors of short‐term tight junction permeability mediators (Rho, ROCK, and MLCK‐dependent actin–myosin contractility) indicated that TNF‐*α* can induce long‐term reorganization of tight junction proteins, although the exact mechanism remains unclear (McKenzie and Ridley 2007). By contrast, to assess the effect of TNF‐induced CD137 expression, the studies reported here used stable expression in transfected cells to test the effects of chronic CD137 expression isolated from other cytokine effects.

Our results highlighted an interesting relationship between CD137 and members of the claudin tight junction protein family. Our data suggest that chronic TNF‐*α* induction of CD137 would induce an increase in claudin‐4 and a decrease in claudin‐3 proteins with no change in other claudins such as 1, 5, and 7. Overexpression of either claudin‐3 or 4 in Caco‐2 cells has been shown to increase TEER, but claudin‐4 may be a more effective mediator of tight junction electrical resistance (Takehara et al. 2009). The explanation may lie in the unique behaviors of each protein; even though claudin‐3 and claudin‐4 have highly homologous sequences, including a highly conserved extracellular loop, they do show differences in lateral associations with other claudins: whereas claudin 3 commonly interacts with CLDN1 and CLDN5 (Daugherty et al. 2007), as well as with claudin‐2, the pore‐forming claudin (Furuse et al. 1999), claudin 4 by contrast prefers only homomeric interactions. Thus, this claudin‐4 homomeric interaction may help explain its potency in increasing tight junction resistance in contrast to claudin‐3.

Our analysis suggests that CD137, anchored at the basolateral plasma membrane, interacts with actin filaments. These CD137 aggregates may function as helpful anchors between the basolateral surface and the basement membrane, as the contact area with the basement membrane may be reduced by the development of the basolateral pocket occupied by interactions with B lymphocytes and dendritic cells. While these aggregates may help stabilize the M‐cell basolateral side, they may also be involved in the interaction between M cells and the basolateral pocket B lymphocytes, which express the ligand CD137L.

Finally, CD137 has been suggested to play role in inflammatory bowel disease in human and mouse models (Maerten et al. 2004, 2006; Lee et al. 2005; Martínez Gómez et al. 2013). Maerten et al. (2004) reported that *CD137* mRNA is upregulated in inflamed Crohn's disease (CD) patient biopsy tissue, although less so in ulcerative colitis (UC). The authors attributed the increased *CD137* mRNA in the biopsy tissue to the immune cell infiltrates, but they did not discuss the possible contribution from mucosal epithelial cells. Interestingly, immunohistochemistry images from the study seemed to indicate that CD137 protein is highly expressed in the epithelial cells, although since detailed analysis of these images (and specificity controls) were not included, we cannot conclude further on this point. Martínez Gómez et al. (2013) offered a possible role for this induced CD137, as they showed that an intact CD137 gene was helpful in the resolution of DSS‐induced colitis in mice. Thus, although CD137^−/−^ and WT mice both had similar disease severity as after 5 days of 3.5% DSS treatment, a distinct difference was detected during a resolution period when DSS exposure was decreased. Here, CD137^−/−^ mice had a significantly increased histology score, number of infiltrating immune cells, and pro‐inflammatory cytokines. Together these observations, along with the results in the present report, suggest that CD137 may play a role in the resolution of intestinal inflammation in part by promoting mucosal barrier tight junction integrity. We do not have information on whether the CD137 in vivo is triggered by CD137L on cells or by extracellular matrix components, but given the apparent global effect of CD137 deficiency, it is possible that ubiquitous ligands such as ECM components may be important. In view of our in vitro studies, we would speculate that the role of CD137 may be through a combination of its influence on claudin protein composition in the tight junctions, and its effects on anchoring the actin cytoskeleton at the basement membrane.

## Acknowledgments

We would like to thank Erinn Parnell for assistance with whole animal fluorescence imaging, Olivia Sakhon for the input on the manuscript. Dr. Chee‐Gee Liew (Duncan) for the generous gift of pCAG‐eGFP‐puro plasmid (Liew et al. 2007), and Kathy Vu, Joseph Pham and Juliane Lieu for assistance with animal husbandry.

## Conflict of Interest

No conflicts of interest, financial or otherwise, are declared by the authors.
